# Formalizing the Function of Anterior Insula in Rapid Adaptation

**DOI:** 10.3389/fnint.2018.00061

**Published:** 2018-12-04

**Authors:** Peter Bossaerts

**Affiliations:** ^1^Department of Finance, Faculty of Business and Economics, The University of Melbourne, Melbourne, VIC, Australia; ^2^The Florey Institute of Neuroscience and Mental Health, Parkville, VIC, Australia

**Keywords:** reference-model based learning, surprise, approximately Bayesian delta-rule learning, Bayesian learning, anterior insula (aIns), risk prediction error, reference model adaptive control (RMAC), active learning

## Abstract

Anterior insula (aIns) is thought to play a crucial role in rapid adaptation in an ever-changing environment. Mathematically, it is known to track risk and surprise. Modern theories of learning, however, assign a dominant role to *signed* prediction errors (PEs), not to risk and surprise. Risk and surprise only enter to the extent that they modulate the learning rate, in an attempt to approximate Bayesian learning. Even without such modulation, adaptation is still possible, albeit slow. Here, I propose a new theory of learning, reference-model based learning (RMBL), where risk and surprise are central, and PEs play a secondary, though still crucial, role. The primary goal is to bring outcomes in line with expectations in the reference model (RM). Learning is modulated by how large the PEs are relative to model anticipation, i.e., to surprise as defined by the RM. In a target location prediction task where participants were continuously required to adapt, choices appeared to be closer with to RMBL predictions than to Bayesian learning. aIns reaction to surprise was more acute in the more difficult treatment, consistent with its hypothesized role in metacognition. I discuss links with related theories, such as Active Inference, Actor-Critic Models and Reference-Model Based Adaptive Control.

## Introduction

Change is a defining characteristic of the human environment. As a result, the need for humans to continuously adapt has made them a most versatile species, whose main skill appears to be the ability to recognize change and learn. Anterior insula (aIns) has long been thought to be a brain structure that is crucial to such rapid adaptation. To recognize that the uncertainty in one’s surroundings has become unusual requires appropriate integration of external sensory signals and ensuing bodily reactions (emotions), while fast behavioral adaptation demands continued awareness, two aspects of learning that evolutionary biologists have attributed to aIns (Craig, 2009, 2011).

Learning signals that have been identified in neural activation within aIns appear to be related to risk and surprise. That is, neural signals correlate with the size of prediction errors (PEs), i.e., the *un-*signed PE (it is always non-negative) (Fouragnan et al., 2017, 2018). aIns neural signals encode the anticipated size of upcoming PEs, which means that they track risk. When uncertainty materializes, aIns neural signals encode surprise, i.e., the extent to which the size of the PE is greater or less than anticipated (Preuschoff et al., 2008).

Yet the dominant model of learning in computational neuroscience is that of reinforcement learning (RL), where surprise does not play a role. Instead, central to RL is the *signed* reward PE (positive if the reward is higher than predicted, negative otherwise). It is this PE which drives updates to estimates of the values associated with stimuli and actions. Encoding of reward PEs has not been associated with aIns; instead, the central role of RL has been attributed to the dopaminergic system, where the reward PE appears to be encoded in phasic firing of dopaminergic neurons of the ventral tegmental area (VTA), as originally discovered in Schultz et al. (1997).

Risk and surprise (risk PEs) at best play an indirect role in RL, in a class of models referred to as approximate-Bayesian delta-rule models (Nassar et al., 2010). There, the learning rate increases, and hence, learning accelerates, when an outcome is dissimilar to outcomes experienced in the past; it decreases if surprise is low (Pearce and Hall, 1980; Behrens et al., 2007; Preuschoff and Bossaerts, 2007; Payzan-LeNestour et al., 2013; McGuire et al., 2014). This modulation makes adaptation faster. One could do without it, at the cost of slower adaptation when outcomes are dissimilar to the past (which would suggest that the environment may have changed), or unnecessary adaptation, when outcomes are similar to the past (which would suggest that the environment remained the same, and hence, learning could gradually be halted). Even in more advanced, model-based forms of learning, PE remains the key variable driving updating (Daw et al., 2005; Gläscher et al., 2010). In model-based RL, the learner has an explicit representation of the potential environments she can be in. The goal is to speed up learning after surprises. This becomes possible since she merely has to identify what environment the new outcomes are most likely to come from; she can thus make more effective predictions after identifying the right environment (Hampton et al., 2006).

As a result, we are left with a puzzle. On the one hand, evolutionary biologists suggest that aIns plays a crucial role in learning and adaptation, yet computational neuroscientists insist on learning models driven by a variable, the PE, that is generally not associated with neural activation in aIns.

The goal of this essay is to propose a solution to this paradox, and to provide provisional evidence of its veracity.

Key to the proposed solution is a novel model of learning, where PE is not the key driver. Instead, learning is meant to bring outcomes as close as possible to predictions from a reference model (RM). Learning increases upon surprises, defined as outcomes that are larger than the RM expected. Learning stops as soon as PEs are in line (or lower than) anticipated in the RM. This leads to novel predictions. In particular, learning may stop even if PEs are non-zero; it will stop if there are no surprises, i.e., the size of the PEs is equal to or less than expected.

In approximately Bayesian delta-rule models, learning is also modulated by the size of the PE relative to expectation. However, such models form these expectations from past outcomes. Learning continues as long as the PEs are non-zero. If the PEs are dissimilar (in size) to recent outcomes, learning may even accelerate. Learning will stop if the PEs remain similar to previous experience, but even then, learning will be reduced only gradually.

The RM could be viewed as encapsulating the notion of ambition and aspirations. As such, the learning model generates “satisficing,” meaning that the learner will be satisfied if she can match the performance of the RM. These notions are absent in the traditional RL model, which aims at optimizing. Psychologists have long criticized this aspect of learning models. Humans, it has been argued, appear to merely spend effort to reach satisfaction, and not to reach optimality. Humans have ambitions and aspirations, will work to reach them, and stop there, even if actions could be improved. Herbert Simon called this form of behavior “satisficing” (Simon, 1957, 1959). But he did not provide a formal model. The learning approach proposed here fills this gap.

The ambition and aspirations are encapsulated in the RM, and the learner is satisfied with her efforts as long as she manages to generate outcomes that are not surprising in view of the model. She could do better (she could still reduce the PE) but if the size of the PE is what is expected in the RM, the learner no longer works toward improving performance.

In this reference-model based learning (RMBL), the PE still plays a crucial role, but it is not central to high-level decisions such as when to start or stop learning, how much effort to spend, whether to explore, etc. That is, PE is not central to metacognition (Fleming and Dolan, 2012); surprise is – relative to the RM.

The remainder of this essay is organized as follows. In the next section, I summarize evidence on identification of mathematical learning signals in aIns. Subsequently, I provide a mathematical account of the proposed RMBL in the context of a task that requires rapid adaptation, involving frequent and salient outliers (“leptokurtosis”). I show that RMBL explains how humans learn in this task. I then position RMBL within the recent literature on learning and control, in engineering as well as in computational neuroscience, and in animal learning. For indeed, the ideas behind RMBL resonate with many aspects of extant modeling. I close with providing perspective, including raising unanswered questions.

## Anterior Insula Anticipates and Tracks Surprise

The first indication that aIns is engaged in tracking surprise came from studies that showed increased correlation of activation with the chance of making mistakes in a task (Klein et al., 2007; Eichele et al., 2008). At the same time, evidence emerged of activation in aIns related to risk and uncertainty (Critchley et al., 2001; Ernst et al., 2002; Paulus et al., 2003; Huettel et al., 2005, 2006). As such, the neural signals revealed a previously unsuspected function of aIns, namely, anticipating the size of PE, or in statistical language, measuring *risk*.

**FIGURE 1:**
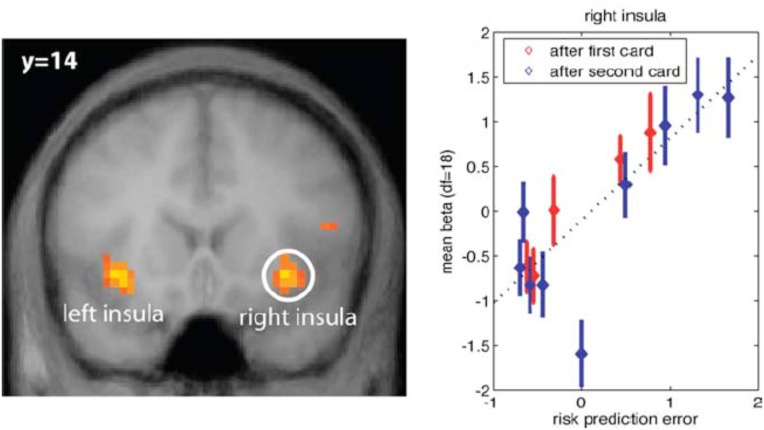
**(Left)** Anterior insula (aIns) activation correlates with surprise, defined as the difference between the size of the prediction error (PE) and the expectation of this size (risk). Results from a card game where potential reward profiles, including expected reward and reward variance (risk) change upon two consecutive draws of cards. **(Right)** Plot of neural activation as a function of risk PE. For control, activation under zero risk, and hence, zero risk PE is shown as well, but not included in the regression line (dotted line) (Preuschoff et al., 2008) (Reproduced by permission #4422210891259).

In addition to risk anticipation, aIns evidently tracks *risk PEs* (Figure 1). These are the difference between the size of the actual PE and its expectation. In other words, it is the unanticipated part of risk, or *surprise*.

There is confusion in the literature, because some would refer to the entire unsigned PE, i.e., the size of the PE, as surprise (see, e.g., Fouragnan et al., 2018). Here, however, we explicitly distinguish between anticipation of the unsigned PE and the mistake in this anticipation. The former is referred to as risk, while the latter is surprise. Interestingly, aIns encodes both the anticipation (risk) and the mistake (surprise).

In the context of reward prediction, aIns activation does not appear to correlate with the (signed) reward PE (Preuschoff et al., 2008; D’Acremont et al., 2009; Fouragnan et al., 2018). This is in contrast to activation in the dopaminergic system, including VTA and its projection regions in striatum (nucleus accumbens) and prefrontal cortex (O’Doherty et al., 2003; Fouragnan et al., 2018). As such, aIns does not encode the key variable that drives belief updating in traditional accounts of learning. Instead, aIns activation appears to focus on the higher statistical moments: risk and surprise.

## A Formal Role for Surprise in Learning

Here, I propose a novel theory of learning which reconciles the importance of surprise in guiding learning, on the one hand, with the traditional view that belief updating ultimately relies on PEs, on the other hand. In this theory, both PEs and surprise about the PE play an important role. Surprise is defined as the magnitude of the PE relative to its anticipation according to a RM. The signed PE is used as in the traditional account of learning, namely, to update forecasts. Surprise is used to change the intensity of learning (i.e., the learning rate).

I present the theory in a simple prediction paradigm. The task is to forecast the next outcome of a sequence of random outcomes generated by a continuously changing underlying state. One can think of the outcomes as a target that moves in space, and the task is to predict the subsequent move (change in location). I will use y(t) to symbolize the location at time (in trial) t (t = 1, 2, …). The underlying, unobservable state is denoted x(t). The observable location y(t) and the underlying state x(t) are related through a traditional state-space model (Dayan and Yu, 2003):

y(t)=x(t)+o(t)

x(t+1)=x(t)+s(t)

Here, o(t) is traditionally referred to as the (random) observation error, and s(t) is known as the (random) state transition. For simplicity, I will assume that all outcomes are scalars, and that the observation error and state transitions are independent from each other and over time.

**FIGURE 2:**
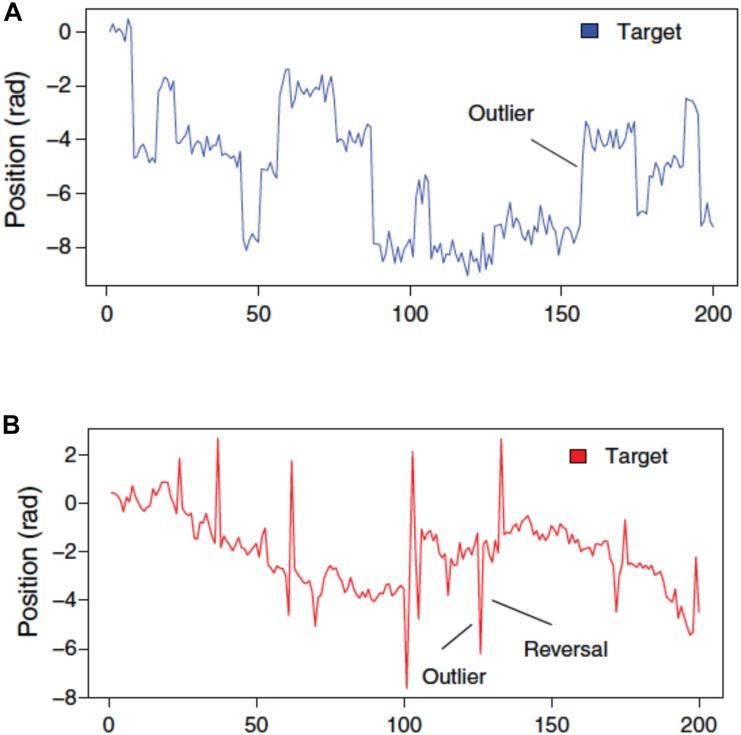
Target movements when state transitions are subject to tail risk **(A)** against when observation error is subject to tail risk **(B)**. **(B)** Outliers therefore revert within one trial (D’Acremont and Bossaerts, 2013) (Reproduced by permission #4422211361599).

Figure 2 illustrates the type of sample paths one can generate with this paradigm. Plot A shows the moves over time of the target when the state is subject to frequent, salient outliers, also known as *tail risk*. Technically, the state transition is governed by a leptokurtic distribution, which is a distribution that is more peaked and has fatter tails than the Gaussian distribution. The observation error, instead, is Gaussian, as usual. In plot B, the observation error displays tail risk: relative to the underlying state, the target sometimes deviates substantially, but this does not have a lasting effect, i.e., the outliers revert. We shall refer to the former as the P treatment, for Permanent (because the outliers have a permanent effect on the location of the target), while the latter will be called the T treatment, for Transitory (because the effect of outliers disappears immediately).

Imagine that the task of our decision-maker, or *agent*, is to predict moves of the target one step ahead. The agent does not know whether she is in the P or T treatment, and hence, forecasting performance will depend crucially on how effectively she learns. If she is told the basic structure of the stochastics driving the target, she could apply full-fledged model-based learning, in the form of Bayesian inference, or use a good approximation based on the delta-rule model. This, however, is very complex. An alternative is to apply (model-free) RL, but one can show that this is extremely slow, and therefore does not match performance of humans, who manage to dissociate between the two treatments within a few trials (D’Acremont and Bossaerts, 2013).

Here, I propose a third possibility. The agent posits a RM against which she evaluates her performance on the task. Imagine that this RM ignores the leptokurtosis, and *instead treats all random outcomes as Gaussian*. As a result, the agent’s RM is a standard Gaussian state-space model. The best forecast of the target location is based on the Kalman filter.

The RM Kalman filter works as follows: the location forecast one step ahead is simply the past location forecast plus a fraction of the present forecast PE. The fraction, referred to as the Kalman “gain,” is fixed over time (provided the filter has reached its steady state). In RL parlance, this gain would be referred to as the learning rate. Significantly, if the observation error and state transitions are indeed Gaussian, the Kalman filter is Bayes-optimal (Meinhold and Singpurwalla, 1983).

Importantly, the RM provides a prediction of the expected size of the forecast error. This expectation remains constant, provided again that the steady state has been reached. Using the square as a measure of size, let Z denote the expected squared forecast error from the Kalman filter.

The goal of the agent is to match the performance of the RM. She is not trying to get the best forecasts, as in Bayesian updating. She only wishes to perform as well as anticipated under the RM. In particular, her forecasts are to be formulated so as to reach the same expected squared forecast error. Surprises (actual forecast errors that are bigger or smaller than expected) lead her to change her forecasting rule.

Imagine that her actual forecasting rule is the same as that in the Kalman filter, *except* that she allows tuning of the gain. That is, her forecasts equal the past forecast plus a fraction of the PE, where this fraction, the “gain”/learning rate, may be changed as a function of surprise. That is, the prediction y^∗^(t) of y(t) equals:

y*(t)=y*(t−1)+G[y(t−1)−y*(t−1)],

where the gain G is to be adjusted in order to minimize the surprise relative to the RM.

The surprise is derived as follows. Let e(t) denote the difference between the actual forecast error in the task and the expected forecast error according to the RM. Since the latter is zero (this is a property of the Kalman filter), e(t) is simply the actual forecast error:

e(t)=y(t)−y*(t),

or:

e(t)=y(t)−y*(t−1)−G e(t−1).

The realized magnitude of the forecast error equals (e(t))^2^. As a result, the surprise (difference between actual outcome and expectation) Ω(t) becomes:

$$\Omega (\text{t})={\left(\text{e}(\text{t})\right)}^{2}-\text{Z}.$$

(Remember, Z denotes the expected squared forecast error from the Kalman filter.) The agent sets the gain in order to minimize the size of the surprise. That is, she chooses G to minimize (Ω(t))^2^:

$${\text{Min}}_{\text{G}}{\left(\Omega (\text{t})\right)}^{2}.$$

G can be found through the first-order conditions for optimality. These imply that G is to be chosen such that:

4Ω(t) e(t) e(t−1)=0.

Generically, the surprise Ω(t) is non-zero. So, the only way the condition can be satisfied is for G to be set so that the product of e(t) and e(t-1) is zero. That is, *the product of the actual forecast error in trial t and t-1 is to be set equal to zero.*

Since this cannot be done trial-by-trial (after all, the forecast error is random), G is to be set so that the *expectation* of this product, i.e., the *autocorrelation* of the forecast errors, is expected to be zero. As a result, the policy adjustment is simple: G is to be changed so that forecast errors become serially independent.

So, if a positive forecast error in trial t-1 tends to be followed by a positive forecast error in trial t (e(t) e(t-1) > 0), forecast adjustments are too timid, and G has to be increased. Conversely, if negative forecast errors in a trial tend to follow positive forecast errors in the previous trial (so e(t) e(t-1) < 0), forecast adjustments are too extreme, and G has to be decreased.

So far, our agent adjusts the gain G in order to minimize the size of the surprise. A more sensible criterion would be to adjust the gain G only if the surprise is negative, i.e., when the size of the actual forecast error is *bigger* than expected. Indeed, if the actual forecast errors are tinier than expected, why change one’s policy? If so, the above gain adjustment policy only kicks in when Ω(t) > 0. (It may be confusing to call surprise negative when in fact Ω(t) > 0, but hopefully this will not create confusion.) Effectively, this means that gain adjustment only happens upon an outlier, i.e., when a forecast error is bigger than expected in the RM.

Summarizing, the adjustment policy is as follows:

• When the size of the forecast error is smaller than expected under the RM, i.e., in the absence of an outlier, keep the gain G constant, at some level G°.
• Upon a forecast outlier, i.e., when the surprise is negative (i.e., Ω(t) > 0), adjust G as follows:
  • Increase G if actual forecast errors are experienced to be positively correlated.
  • Decrease G if actual forecast errors are experienced to be negatively correlated.

**FIGURE 3:**
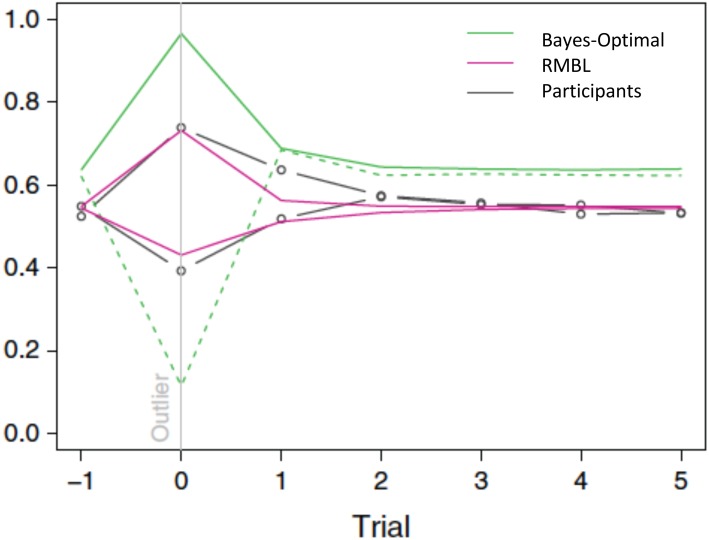
Gain (learning rate) around outlier trials (outlier = target moves more than 1 standard deviation). Green: Bayes-optimal gain in P (solid line) and T (dashed line) treatments. Magenta: RMBL gain in P (line moving up in outlier trial) and T (dashed line moving down) treatments. Black: average participant gains, stratified by treatment. As in the RMBL model, participants’ gain upon an outlier in the T treatment is about three times higher than optimal (D’Acremont and Bossaerts, 2013) (Reproduced by permission #4422211361599).

Interestingly, this appears to be exactly what humans do (D’Acremont and Bossaerts, 2013). The above policy fits human forecasts in the location prediction task. In particular, it explains why, in the T treatment (where the observation error is leptokurtic), humans tend to under-estimate the reversals: their forecasts only partially adjust for the fact that large movements in the target constitute mostly noise, and hence, should be discounted when forecasting the subsequent location. The gain they apply is about three times higher than the Bayes-optimal gain. In the P treatment, it is closer to the Bayes-optimal gain: it equals 80% of the Bayes-optimal gain. See Figure 3. As a result of differential policies in the treatments, humans do much better in the P than in the T treatment.

In our example of RMBL, both forecast errors and surprise (unanticipated sizes of forecast errors) play a direct role in policy adaptation. In a delta-rule based RL that approximates the Bayes-optimal policy, surprise also plays a role in modulating the learning rate. There, however, surprise is defined differently: it obtains as a result of a comparison of the size of the current forecast error with the typical size of previously experienced forecast errors. In RMBL, surprise is defined as the difference between the size of the latest forecast error and the prediction (of this size) according to the reference Kalman filter model. Figure 3 demonstrates that approximately Bayesian delta-rule learning does not match human choices as well as the RMBL.

**FIGURE 4:**
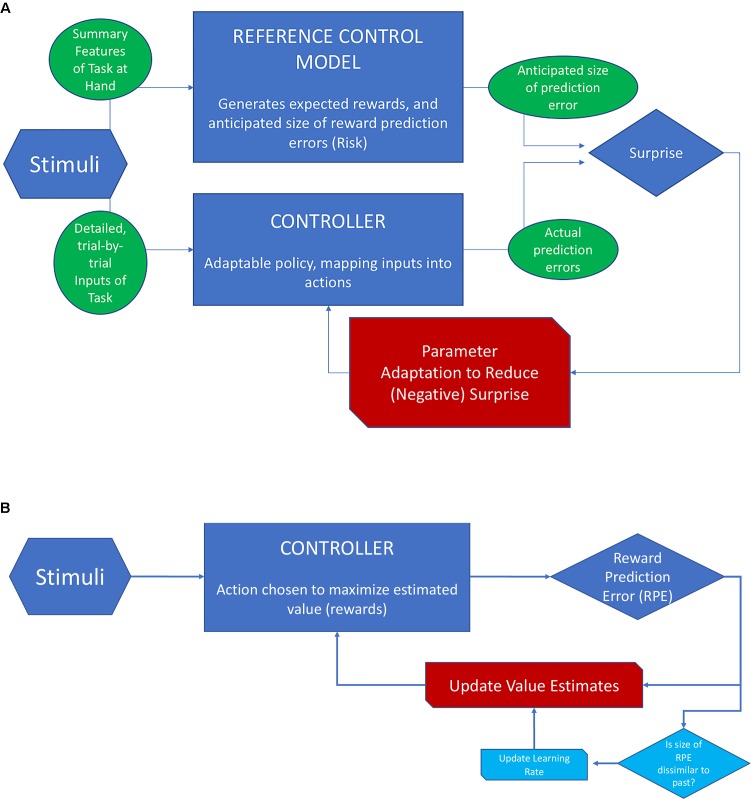
Schematic view of RMBL and RL. **(A)** In RMBL, policy parameters are altered indirectly in response to surprise (red channel), defined as the difference between the size of the PE and its expectation according to a reference model (RM). RM encapsulates the agent’s analysis of the task at hand, including reward and PE profiles that she expects to be achievable. **(B)** In RL, the controller optimizes actions based on estimates of values (sum of expected future rewards). Upon an outcome, the PE is used to update value estimates (red channel). In approximately Bayesian delta-rule models, the updating speed (gain/learning rate) is adjusted as a function of dissimilarity of the size of the PE relative to recently experienced PEs (channels in light blue).

Figure 4 provides a schematic overview of RMBL and contrasts it with the scheme for RL when applied to reward prediction (as opposed to location prediction). In RL (Scheme B), the goal is to minimize the error of value estimates (values are the sum of future rewards), and the action is chosen to maximize value. Value estimates are updated based on the history of PEs. In approximately Bayesian delta-rule learning, the speed of updating depends on a comparison between the size of the last PE and that of previously experienced PEs. In RMBL (Scheme A), the RM takes basic features of the stimuli to identify the task, generates expectations of rewards that could be reached, and estimates of the size of reward PEs. The latter could be conceived as an estimate of the uncertainty that is left after controlling the environment. The (separate) controller then takes detailed inputs from the stimuli, chooses an action based on a policy (that can be adapted), and generates a reward. This reward is compared to its expected value according to the RM, which produces a PE. The size of the PE is compared to the reference-model based anticipation, which produces a surprise. If this surprise is large (the size of the PE is bigger than anticipated), the policy is adapted.

## Antecedents of Reference-Model Based Learning

In many respects, the idea of minimizing the size of surprise relative to a RM has appeared in the literature before. That is, RMBL is not fundamentally new.

It resonates well with *active inference* (Rao and Ballard, 1999; Friston et al., 2009, 2012; Friston, 2010), whereby the agent attempts to act upon the environment in a way that brings outcomes closer (in distribution) to prior expectations. In active inference, the agent minimizes the distance between the distribution of outcomes generated through control of the environment and what she expects this outcome distribution could be. Here, surprise is the distance between two distributions: it is the distance between a distribution that is aspired to, and the distribution of the actual outcomes. The aspired distribution remains fixed, in analogy with the RM in RMBL.

The *actor-critic model* of RL (O’Doherty et al., 2004) also shares some commonalities. There, one can think of the critic as the RM, and the actor as the controller. The main difference with RMBL, however, is that in RL the critic constantly updates its expectations based on what the actor managed to generate in the past. In RMBL, the model is autonomous.

Reference-model based learning is related to *reference model adaptive control* (RMAC) in engineering (Nguyen, 2018). This approach to control has been advocated for situations where the environment is too complex to be modeled directly, or aspects of the environment change too quickly or remain unknowable. RMAC was suggested as an alternative to robust control, which tends to choose actions to guard only against the worst-case scenario, thereby foregoing huge opportunities in case the environment is more benign than expected. Like in RMBL, optimality is not aimed at; adaptability is, instead. Ensuing controls are “satisficing,” not optimizing. Control is satisfactory as long as PEs are in line with the RM; no further optimization is aimed at.

The specific model that our example generated, whereby forecasts are adjusted when forecast errors exhibit autocorrelations, is reminiscent of work by Sutton (1992). In his case, however, the adjustment policy is exogenously imposed and does not emerge from a desire to minimize surprise relative to a RM.

Reference-model based learning makes assumptions about the prior an actor will bring to a circumstance, in the form of a RM. Bayesian updating also starts with a prior. But there is a crucial difference. In Bayesian updating, the model (prior) changes with experience, and the goal is to ultimately recover the truth. In most accounts of Bayesian modeling, the truth is ultimately recovered, and hence the prior model is irrelevant. [It is little appreciated that this is not generic (Diaconis and Freedman, 1986); but that need not concern us here.] In RMBL, the model is *not* updated (it may in the long run; see below). Because the prior ultimately has no influence on learning in standard Bayesian paradigms, one could dispense with it, and this explains why delta-rule models can be almost as good as Bayesian, or “approximately Bayesian” even if they do not start from a prior (Nassar et al., 2010).

As a result, RMBL makes very different behavioral predictions from Bayesian learning or its delta-rule approximations. To highlight the difference, consider a simple task where participants have to adjust either to a (single) hidden shift in mean of the outcome distribution, or to a shift in the variance. Imagine that, in the latter case, the variance is increased. Both the mean shift and the variance shift generate marked outliers given prior experience. Consequently, Bayesian learning, or approximately Bayesian delta-rule learning, will increase the learning because of the outlier. Therefore, beliefs quickly adjust to the distributional shift.

In our RMBL model updating only happens when forecast errors are significantly auto-correlated, not when outliers occur. As a result, when the mean shifts, predictions are adjusted, because PEs become (positively) autocorrelated: either one constantly over-estimates subsequent outcomes, or one under-estimates them. So, under mean-shifts, adjustments take place. In contrast, when only the variance increases, PEs remain uncorrelated over time, and there is no adjustment. Learning does not take place!

Interestingly, the latter account appears to be consistent with the evidence (Filipowicz et al., 2018): human participants learn effectively in mean-shift paradigms, while they adapt less rapidly in variance-shifts paradigms.

## Perspective

I proposed a formal modeling approach to learning, RMBL, that reconciles the allegedly crucial role of aIns in rapid adaptation, on the one hand, and the centrality of PEs in learning, on the other hand. Reconciliation is called for since aIns activation does not reflect PEs. Instead, neural signals reflect the anticipated size of PEs and the subsequent surprise if the size is different from anticipated. In RMBL, anticipation is not based on experience prior to the surprise, but on a RM that remains rigid throughout learning.

In the approach, the agent does not attempt to optimize, as in traditional accounts of learning such as RL or Bayesian optimization and its delta-rule approximations. Instead, she works to bring outcomes as close as possible to predictions from her RM. Instead of maximizing expected value, she minimizes surprise, defined as the difference between the PE generated in the environment and the expected PE as anticipated in her RM. One would want her to minimize surprise only if it is larger than anticipated, which is what I proposed in the analysis of the location forecasting paradigm.

The RM encapsulates what the agent ideally expects to obtain through control of her environment. Actual control of the environment should match these expectations in the sense that surprises are minimized. The agent does not optimize. She merely “satisfices” (Simon, 1957, 1959). The RM reflects her ambition and her aspiration; if she can match it, she is content. This is in sharp contrast with Bayesian learning and its delta-rule approximations, where the goal is to attain control that is optimal given the environment.

Evidence of “satisficing” behavior has emerged, among others in a lesion-patient study on the game “rock-paper-scissors” (Danckert et al., 2012). In this game, the best response to an opponent who does not mix uniformly as in the Nash equilibrium is to switch to a pure strategy and choose the particular option that wins from the option that the opponent chooses most frequently. Interestingly, healthy controls do not do so. Instead, they merely match the probabilities of the opponent. As a result, their payoffs are less than when optimizing. It is as if participants were satisfied with disequilibrium payoffs that only slightly improve on the mixed-strategy Nash equilibrium, where the player wins with 50% chance; it is as if their “reference model” was the Nash equilibrium, and once they beat it (profits are higher than anticipated), they stopped learning.

In contrast, left-hemisphere lesion patients, *especially those with lesions in Insula*, optimize: they switch to best-responding (e.g., “rock” if the opponent mostly plays scissors). With insula lesions, PEs appear to no longer be evaluated against a RM that expects Nash equilibrium payoffs. This leaves the patients only with evaluation against recent experience. Paradoxically, this eventually makes them optimizers, rather than “satisficers.”

Why would one use a rigid RM in learning? I already mentioned its motivation in engineering: robust control. But RMBL may also facilitate metacognition. If the agent is to perform multiple tasks at once, it is important that she pays attention only to tasks that require adjustment, while leaving the remaining tasks to automatic (habituated) execution. A quick way to determine adaptation need is to compute surprise relative to a rigid RM. It obviates the need to constantly update priors as in Bayesian learning, for each task, including those not attended to. Attendance then boils down to fine-tuning policy (action profiles) only for tasks that generate negative surprise relative to their respective RMs. The remaining tasks are left unattended; they are executed using learned (habituated) policies.

This model of metacognition squares well with the alleged role of aIns in awareness (Craig, 2009). As mentioned before, neural activation in aIns reflects risk and surprise. At the same time, proper functioning of aIns has been found to be crucial for awareness. Awareness is needed when surprise occurs, i.e., when risk is higher than anticipated. The conjecture is that aIns contributes to integrating tracking of surprise and allocation of attention.

In our target location forecasting task, participants performed worse in the T treatment, and hence this must have generated bigger surprises. Proper reaction to reverting outliers required increased vigilance. The finding that aIns activated significantly more vigorously upon outlier trials in the T treatment is consistent with aIns’ role in metacognition (Fleming and Dolan, 2012). The increased reaction times in the T treatment are also consistent with this.

Finally, a natural question still needs answering: where does the RM come from? In engineering, RMs are chosen to ensure robust control. In human learning, one can imagine that its goal is the same. Presumably, this requires that the RMs are updated as well, but at a slower rate. That is, in case the agent is unable to avoid negative surprises (she cannot control her environment as much as she aspires too; the RM is no longer robust), the RM needs updating. Conversely, if the agent experiences predominantly positive surprises (her RM constantly under-estimates how much her environment can be controlled), ambition may be changed through adaptation of the RM. I leave it to future work to explore how and how fast this occurs.

Whatever the mechanism behind updating of the RM, inability to adapt it when needed may be a plausible way to explain symptoms of mental disorders. Depression and anxiety may be the consequence of inability to avoid negative surprises. Compulsive behavior may be the result of continuous positive surprises. Symptoms could be alleviated by decoupling learning and the RM, through cognitive-behavioral therapy, deep-brain stimulation or medication. RMBL may thus provide fresh insights into, and remediation of, maladaptive behavior.

## Author Contributions

The author confirms being the sole contributor of this work and has approved it for publication.

## Conflict of Interest Statement

The author declares that the research was conducted in the absence of any commercial or financial relationships that could be construed as a potential conflict of interest.
